# Editorial: Neural circuits and neuroendocrine mechanisms of depression and premenstrual dysphoric disorder: towards precise targets for translational medicine and drug development, volume II

**DOI:** 10.3389/fpsyt.2023.1216689

**Published:** 2023-06-13

**Authors:** Sheng Wei, Yi-Yuan Tang, Fushun Wang, Yang Wang, Xiwen Geng

**Affiliations:** ^1^Experimental Center, Shandong University of Traditional Chinese Medicine, Jinan, China; ^2^College of Health Solutions, Arizona State University, Tempe, AZ, United States; ^3^Institute of Brain and Psychological Science, Sichuan Normal University, Chengdu, China; ^4^Department of Integrative Medicine, Xiangya Hospital of Central South, Changsha, China

**Keywords:** major depressive disorder, premenstrual dysphoric disorder, neural circuits, neuroendocrine, translational medicine

Premenstrual syndrome (PMS), along with one its severe subtypes, premenstrual dysphoric disorder (PMDD), significantly affects the physical and mental healths of the women of reproductive age (1). Common symptoms in the women that are susceptible to these disorders include emotional problems, such as irritability, depression, anxiety, emotional instability, anhedonia, and lassitude, as well as physical problems, such as breast tenderness, weight gain, distension, muscle and joint pain, headache, and limb edema (2), that are considered to be an exaggerated and inappropriate response to everyday acute stressful challenges (3). Major depressive disorder (MDD), in contrast, is the most common mental illness in modern society. Clinical features include significant and persistent depression or loss of interest, as well as other symptoms, such as loss of self-esteem, inappropriate feelings of guilt, suicidal ideation, and impairment of cognitive function. The physical symptoms include symptoms, such as sleep, appetite, and sexual behavior, with a chronic and recurrent course (4). MDD and PMDD are the two common types of depressive disorders described in the diagnostic and statistical manual of mental disorders (5). Depressive disorder, represented by PMDD and MDD, is the most common mental illness in modern society (4). Depressive disorders have become one of the most serious diseases threatening human health due to socioeconomic development, competitive pressures, unemployment, and changes in the pace of life. Depressive disorders are characterized by high prevalence, relapse rate, high disability and death rate, and is known as the “number 1 psychological killer”. The World Psychiatric Association survey shows that the global prevalence of depression is 4.2 and 6.9% in case of China, with an annual growth rate of 113%. Global Burden of Disease data shows that mental/neurological disorders account for the largest burden of disease, with depression accounting for the largest share of mental/neurological disorders (6).

There are similarities between the symptoms of PMDD and MDD. Most women of reproductive age experience varying degrees of physiological and psychological discomfort before menstruation begins. These symptoms peak ~1 week before menstruation and improve or disappear after menstruation. When these symptoms reach a level that interferes with daily life, they are called PMS. Previous studies have shown that PMS and its severe subtype PMDD have a high comorbidity with other mood disorders, of which depression is the most common (7). For instance, 22% of the women with PMS also suffer from major depression, while another 5.4% show mild depressive symptoms (8). A 2-year longitudinal study showed that the women with PMS develop depression at a rate 14 times higher than that of healthy women (9). Given the high comorbidity of PMS and depression, many researchers believe that PMS should be considered a variant of depression (10). Empirical findings also suggest that many risk factors are shared between PMS and depression, such as early childhood abuse, unhealthy lifestyles, non-adaptive emotion regulation strategies, abnormal emotional response, stress response, and reward processes (11). However, other researchers have emphasized that although there are many similarities between PMS and depression, PMS should be considered a distinct diagnostic entity rather than a variant of depression because its most salient features are irritability and mood instability, rather than a depressed state of mind (12). Empirical studies have also found differences in the abnormalities in stress responses, rewarding processes in women with PMS and depression (10). Numerous studies have reported a positive association between premenstrual changes and psychiatric disorders, especially depression. Patients with depression often have worsening symptoms in the premenstrual period and are often admitted to hospital in the premenstrual period. There is a significant increase in attempted suicides in the premenstrual period and ~57% of the women with a lifetime diagnosis of MDD have premenstrual depression. Hence, premenstrual irritability changes are an indicator of vulnerability to MDD (13). In conclusion, it is difficult to distinguish and identify female depression and PMDD patients etiologically and symptomatically, since both perhaps share a common pathogenesis as both are closely related to neurocircuitry and neuroendocrine abnormalities (14).

Till now, the pathogenesis of depression has not been fully elucidated. The current treatment drugs includes mainly the selective serotonin reuptake inhibitors. However, 30–40% of the patients suffering from depression are not sensitive to the drug treatment and have significant psychological side-effects, slow onset, and some patients may even develop drug resistance, with obvious time lag and low efficacy (15). Although single-target chemicals can exert a better “magic bullet effect”, the therapeutic effect is not ideal. Therefore, the advantages of traditional Chinese medicine, which can exert intervention effects through multiple targets and links, is highlighted. The core pathogenesis of depressive disorders is the neural circuit and neuroendocrine, and the drug effects acting on these two pathways are significantly different. Based on the available findings, we speculate that the rapid onset of antidepressants is regulated by the neural circuit, while the sustained onset is regulated by the neuroendocrine system, which is the hypothesis of rapid and sustained onset of antidepressants action mechanism (16–19). We further infer that as a multicomponent system, some traditional Chinese medicines contain both components that act on the neural circuit and those that act on the neuroendocrine system, thus acting on the both pathways simultaneously. Therefore, it can effect quickly and maintain it over a longer period (Figure 1). In recent years, this hypothesis tends to be confirmed by the excellent research work of many scholars. A systematic and in-depth work around this hypothesis can better explain the neurobiological mechanisms of antidepressant effects, providing valuable ideas and clues for finding new antidepressants that can effect quickly and maintain it over a longer period of time.

**Figure 1:**
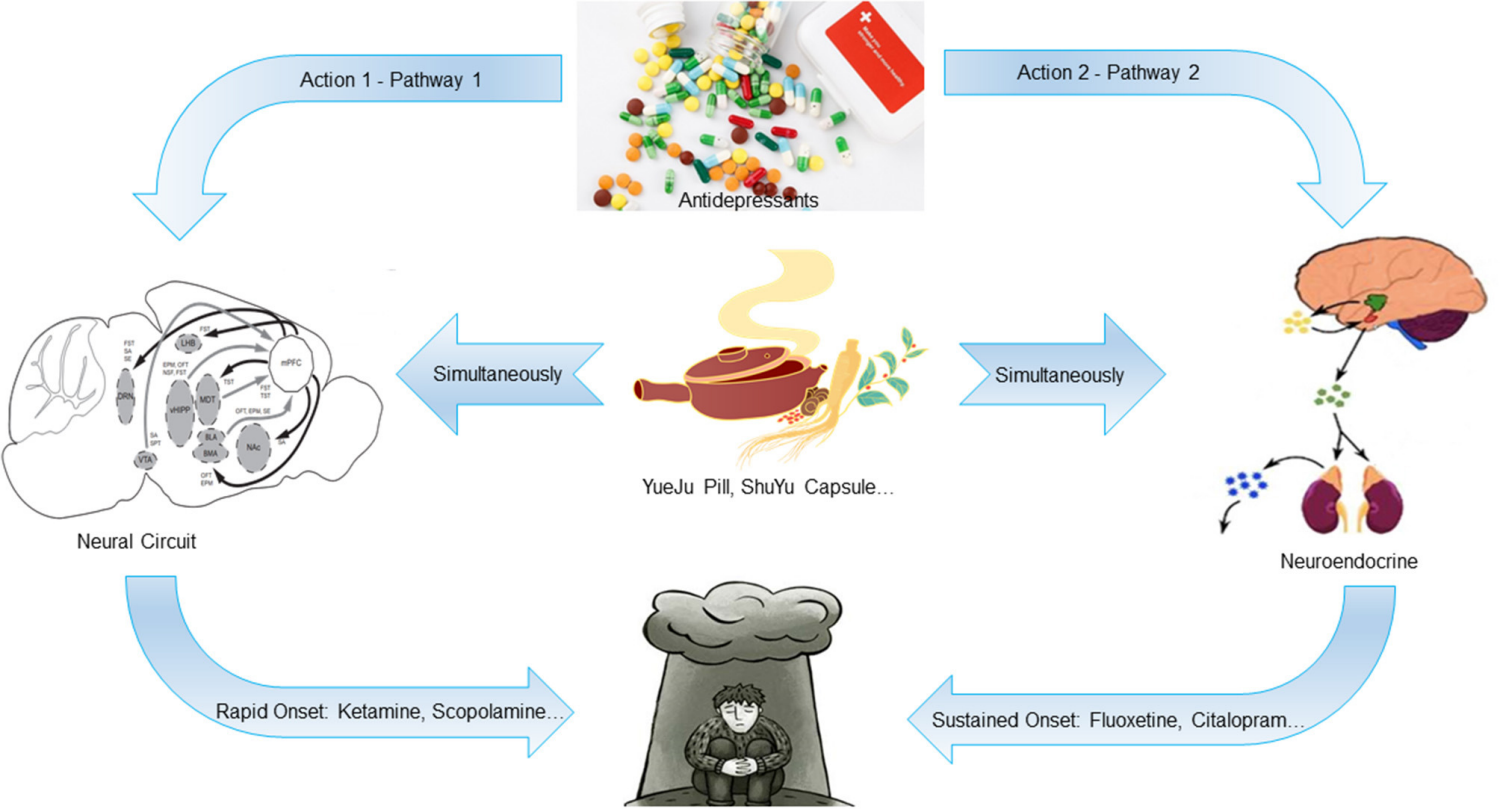
Schematic diagram of the hypothesis of rapid and sustained onset of antidepressants action mechanism. The rapid onset of antidepressants is regulated by the neural circuit, while the sustained onset is regulated by the neuroendocrine system. Some traditional Chinese medicines contain both components that act on the neural circuit and those that act on the neuroendocrine system, which can act on both pathways simultaneously. Therefore, it can take effect quickly and maintain a longer time.

Based on the above basic considerations, we organized this Research Topic to deeply understand the neural circuit and neuroendocrine mechanism of PMDD and MDD, especially the possible intervention targets and mechanisms of antidepressants and traditional Chinese medicine. This effort attempts to provide referential ideas and directions for the research and development of new antidepressant drugs toward precise targets and translational medicine. Therefore, we invited commentary or research papers in this field. About 14 submissions were received, and after nearly 10 months of peer review, 6 papers were successfully accepted.

In the paper titled “*Depression in polycystic ovary syndrome: focusing on pathogenesis and treatment*,” Xing et al. discuss the epidemiology of depression in polycystic ovary syndrome (PCOS) and potential pathogenic mechanisms underlying PCOS and depression. Some of the common treatment strategies for depression in PCOS are also reviewed in this study.

In Zhang et al.'s paper, titled “*Multi-level variations of lateral habenula in depression: a comprehensive review of current evidence*,” the authors systematically combed advances from rodents, summarized changes in the lateral habenula and related neural circuits in depression, and attempted to analyze the intrinsic logical relationship among these pathological alterations.

In the paper titled “*Role of allopregnanolone-mediated* γ*-aminobutyric acid A receptor sensitivity in the pathogenesis of premenstrual dysphoric disorder: Towards precise targets for translational medicine and drug development*,” Gao et al. described the emotional regulatory effect of allopregnanolone (ALLO), summarized the relationship between ALLO and γ-aminobutyric acid A (GABA_A_) receptor subunits and discussed in depth the treatment of PMDD with targeted GABA_A_ receptors, hoping to find a precise target for drug development and subsequent clinical application.

In the experimental report titled “*The relationship between liver function and neurophysiological factors in depressed individuals: a cross-sectional study using an integrated ‘East meets West' medicine approach*,” Ye et al. conducted a cross-sectional study among 100 participants in a rehabilitation hospital and found that traditional Chinese medicine-based liver function can be interpreted using the hypothalamic-pituitary-adrenal axis.

In another study titled “*The opposite effects of estradiol and progesterone on woman's disgust processing*,” Liu et al. performed behavioral and resting-state functional magnetic resonance imaging studies on the effects ovarian hormones on disgust emotion, and found that a more negative attitude to disgust stimuli and the enhanced functional connectivity of the salience network during the luteal phase may be associated with high progesterone levels, whereas lower disgust feelings and reduced functional connectivity of the amygdala during the follicular phase may be associated with high estradiol levels.

In Han et al.'s paper titled, “*Neuroendocrine pathogenesis of perimenopausal depression*,” the authors elaborate the neuroendocrine mechanism of perimenopausal depression from the aspects of epigenetic changes, monoamine neurotransmitter and receptor hypothesis, glial cell-induced neuroinflammation, estrogen receptor, interaction between the hypothalamic-pituitary-adrenal and hypothalamic-pituitary-gonadal axes, and microorganism-brain gut axis.

In summary, the papers accepted in this Research Topic discuss the extensive neural circuits and neuroendocrine mechanisms of PMDD and MDD from different perspectives. It is particularly interesting that two papers used clinical subjects to explore the impact of hormones on adverse emotions and the manifestation of traditional Chinese medicine-based liver function, demonstrating the breadth and depth of the content included. The widespread attention to the neural circuits and neuroendocrine mechanisms of PMDD and MDD reflects the forefront and direction of this field. We are convinced that with more experimental work to verify the hypothesis of rapid and sustained onset of antidepressants action mechanism proposed above, our understanding of the pathogenesis and intervention mechanism of PMDD and MDD will be more profound, which will greatly promote drug development based on precise targets and translational medicine, and provide referential ideas and methods for solving the sufferings of the patients.

## Author contributions

SW, Y-YT, FW, YW, and XG wrote the paper. All authors agreed on publishing the manuscript.
